# Supplementary material for: Chemical genetics and proteome-wide site mapping reveal cysteine MARylation by PARP-7 on immune-relevant protein targets
Source: eLife. 2021 Jan 21;10:e60480. doi: 10.7554/eLife.60480 (PMC7880690; doi:10.7554/eLife.60480)
Supplement: Figure 3—source data 1. — Phthal01 (6-(4-(2-fluoro-5-((4-oxo-3,4-dihydrophthalazin-1-yl)methyl)benzoyl)piperazin-1-yl)nicotinonitrile) was tested against all active PARP family members using an ADP-ribosylation plate assay developed in our lab (Kirby et al., 2018). IC50 Data represents ± SEM from at least two biological replicates. [file elife-60480-fig3-data1.docx]

| **Enzyme** | **IC50 (µM)** |
| --- | --- |
| PARP1_FL_ | 0.021±0.004 |
| PARP2_FL_ | 0.028±0.005 |
| PARP3_FL_ | 0.32±0.002 |
| PARP4_brct-cat_ | 0.35±0.002 |
| PARP5b_cat_ | 2.52±0.2 |
| PARP6_FL_ | 0.18±0.05 |
| PARP7_FL_ | 0.014±0.002 |
| PARP8_FL_ | 4.7±1 |
| PARP10_cat_ | 0.86±0.3 |
| PARP11_FL_ | 0.46±0.02 |
| PARP12_cat_ | >3 |
| PARP14_wwe-cat._ | >10 |
| PARP15_cat_ | 2.0±0.3 |
| PARP16_FL_ | 4.3±1 |

**Figure 3—source data.** *In vitro* IC_50_ data for Phthal01 against PARP family members. Phthal01 ( 6-(4-(2-fluoro-5-((4-oxo-3,4-dihydrophthalazin-1-yl)methyl)benzoyl)piperazin-1-yl)nicotinonitrile) was tested against all active PARP family members using an ADP-ribosylation plate assay developed in our lab (Kirby *et al.* 2018). IC_50_ Data represents ± SEM from at least two biological replicates.
